# Right bundle branch block evolving to myocardial ischemia in a patient with chronic middle back pain: a case report

**DOI:** 10.1186/s13256-023-03842-z

**Published:** 2023-04-06

**Authors:** Meity Ardiana, Inna Maya Sufiyah, Muhammad Nuh Hamdani

**Affiliations:** Cardiology and Vascular Medicine Department, Medical Faculty of Airlangga University-Dr. Soetomo General Hospital, Surabaya, Indonesia

**Keywords:** Right bundle branch block, Myocardial infarction, Back pain, Atypical angina

## Abstract

**Background:**

A right bundle branch block (RBBB) is rarely found in patients with myocardial infarction (MI). In addition, back pain is an atypical complaint in patients with angina.

**Case:**

A 77-year-old Javanese male was admitted with middle back pain that he had had for several months but that had become worse in the past week. He received an oral nonsteroidal anti-inflammatory drug as analgesic therapy but the pain did not improve. The patient came to the emergency room and an electrocardiogram (ECG) showed complete RBBB and first-degree atrioventricular block. Three days after hospital admission, his chief complaint of pain had worsened, and ECG showed new deep arrow-head inverted wave at V3–V6, II, III, and aVF, as well as infero-anterolateral ischemia. The coronary angiography revealed 95% critical stenosis in left circumflex artery.

**Discussion:**

It is a challenge for clinicians to recognize and carefully assess a patient’s complaints even if they are admitted for pain that is “atypical” of MI. When ECG shows changes, clinicians need to pay attention to a tricky, hidden, and life-threatening occlusion of the coronary artery.

## Introduction

A right bundle branch block (RBBB) is characterized by a lengthening of the QRS duration by more than 120 ms and an rsr', rsR', or rSR' pattern in V1 or V2 of the right chest. A review of the published literature reveals a dearth of articles on how to interpret electrocardiography (ECG) abnormalities caused by an infarct or an ischemic event when there is RBBB [1]. It only appears in about 6% of cases of myocardial infarction (MI) [2]. The latest European Society of Cardiology guidelines describe RBBB as a high risk for mortality in patients with suspected MI [3]. Back pain is a rare complaint in patients with suspected coronary disease. According to Patel [4], the severity of back pain and the mortality rate are correlated with coronary heart disease. This case report presents a patient with back pain and RBBB on ECG who progressively developed MI.

## Case presentation

**Fig. 1:**
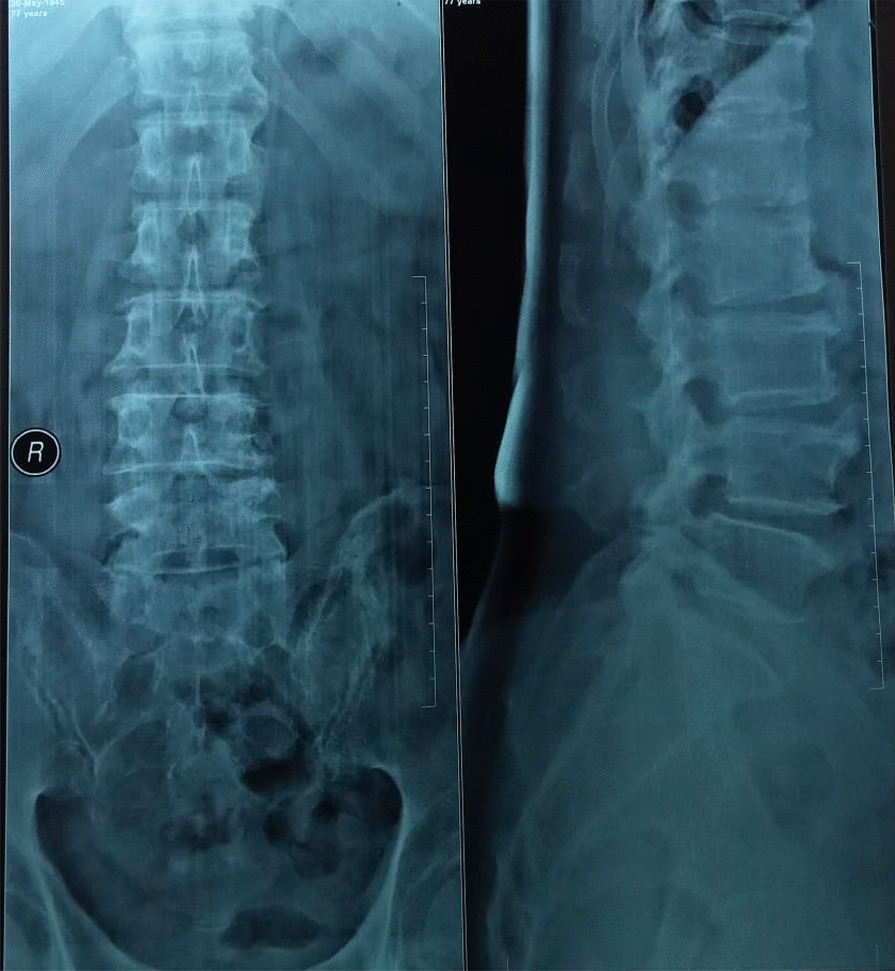
Lumbar X-Ray depicting spondylolithesis of lumbar vertebra 4–5

A 77-year-old Javanese male was admitted for middle back pain; he had had the pain for several months, but it had gotten worse in the past week. He described his recurrent back pain as feeling heavy pressure in the mid-back; sometimes it spread to the back of the epigastric region. He had felt fatigued in the previous 2 weeks, with dyspnea, palpitation, perspiration, nausea, and vomiting but without chest pain. His medical history included hypertension and diabetes mellitus for the past 20 years. He was regularly taking amlodipine 10 mg once a day for hypertension, and metformin 500 mg three times a day and glimepiride 2 mg once a day for diabetes. The patient denied any other history of coronary heart disease or kidney disease. He had quit smoking 5 years ago and had never drank. He had no family history of cardiovascular and metabolic disease. A lumbar X-ray was performed (Fig. 1) and the patient received an oral non-steroidal anti-inflammatory drug (NSAID) as analgesic therapy but he did not improve.

**Fig. 2:**
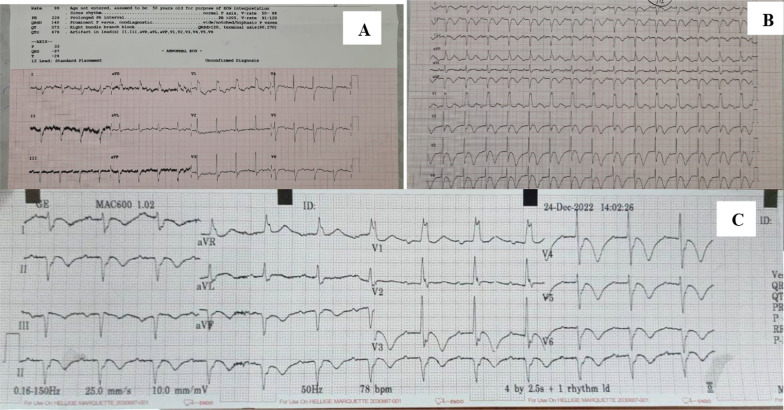
The electrocardiogram (ECG) evolution of the patient. **A** The first ECG when the patient came to emergency room complaining of heavy pressure in his back; it showed inferior old myocardial infarction (OMI), complete right bundle branch block (RBBB), and first-degree atrioventricular block. **B** The patient returned with greater back pain, and the next ECG during hospital admission showed new deep arrowhead inverted T waves at V3–V6, II, III, and aVF; inferior OMI; complete RBBB; and first-degree atrioventricular block, with heavier of back pain. **C** The third ECG was done in the emergency room of our referral hospital; it showed progression of the ischemic signs from the previous ECG, without improvement in the chief complaint

The patient then came to the emergency room of a hospital and the ECG showed the QS pattern at II, III, and aVF; inferior old myocardial infarction (OMI); complete RBBB; and first-degree atrioventricular block (Fig. 2A). After being admitted, his back pain did not decrease with analgesic injection three times a day. By day 3 of hospital admission, his chief complaint (pain) had become burdensome: His back pain was so bad that he could not sleep on his back. The ECG on day 3 showed new deep arrowhead inverted T waves at V3–V6, II, III, and aVF; signs of infero-anterolateral ischemia and inferior OMI; complete RBBB; and first-degree atrioventricular block (Fig. 2B). Hence, the patient was referred to our hospital, which has a percutaneous coronary intervention (PCI) facility.

In the emergency room of our hospital, as the referral hospital, we re-measured his vital signs. His blood pressure was 120/82 mmHg, his heart rate was 79 beats per minutes (normal range 60–100 beats per min), his respiration rate was 18/min (normal range 12–20/min), and his oxygen saturation was 98% on free air (normal range 95–100%). His body weight was 56 kg and his height was 170 cm (body mass index = 19.3 kg/m^2^). There were no positive physical examination findings. All other laboratory values were within normal range except for the white blood cell (WBC) count of 13.55 × 10^3^/uL (normal range 3.37–10.0 × 10^3^/uL), the neutrophil count of 81.2% (normal range 39.8%–70.5%), and the cardiac marker high-sensitivity troponin I level of 996.5 (normal range < 35). We performed an ECG (Fig. 2C) and there was no improvement, showing persistent deep arrowhead inverted T waves at II, III, aVF, and V3–V6; inferior OMI; complete RBBB; and first-degree atrioventricular block.

**Fig. 3:**
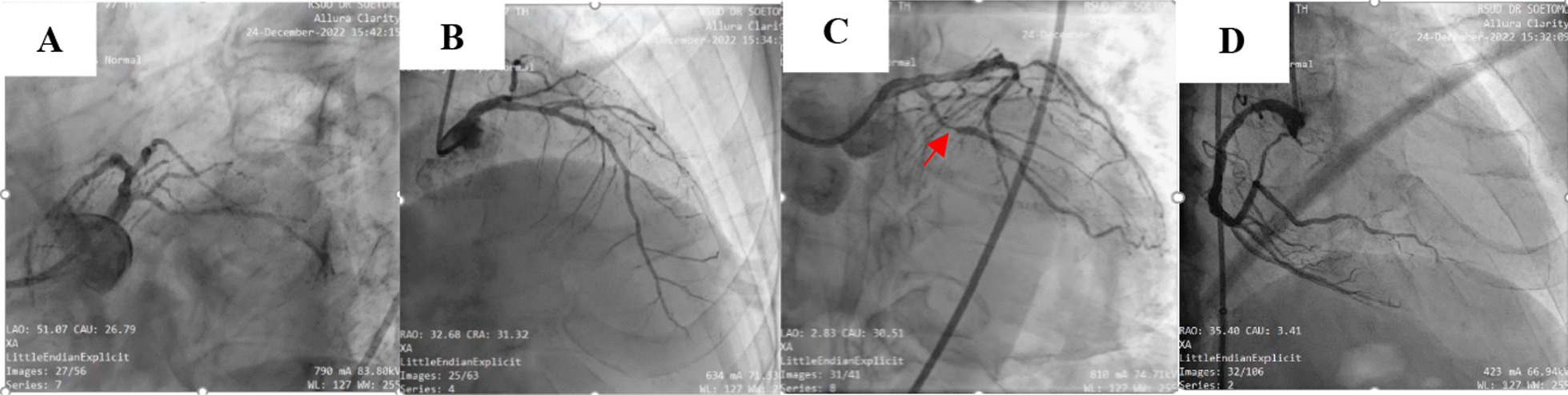
Coronary angiography results of the patient: **a** normal left main coronary artery (LMCA); **b** diffuse disease with maximal stenosis of 30% at the proximal distal end of the left anterior descending artery (LAD); **c** the red arrow shows diffuse disease, with maximal critical stenosis of 95% at the distal end of the left circumflex artery (LCx) and Thrombolysis in Myocardial Infraction Flow III; **d** non-significant 30% stenosis of the right coronary artery (RCA)

There was also a decrease in left ventricular systolic function (ejection fraction: 46%), and third-degree left ventricular diastolic dysfunction based on echocardiography. Hence, he underwent urgent coronary angiography (Fig. 3). It revealed 95% critical stenosis in the left circumflex artery (LCx), 30% stenosis in the mid-left anterior descending (LAD) coronary artery, and non-significant stenosis (30%) in the mid right coronary artery (RCA).

The patient was subsequently managed with a primary percutaneous coronary intervention (PPCI) because of the high risk for non-ST elevation acute coronary syndrome (single vessel coronary artery disease + 95% critical stenosis of the distal LCx) and Thrombolysis in Myocardial Infraction (TIMI) Flow III. After placing the stent, the patient said that his back pain improved dramatically. He received an antiplatelet drug, nitrates, an angiotensin-converting enzyme inhibitor, a statin, and a beta-blocker. During follow-up the back pain had disappeared so he did not need an analgesic drug. The prognosis for this patient is good because he has single vessel disease and the stent was successful.

## Discussion

Our patient’s case represents a rare life-threatening and unpredictable situation. His chief complaint of mid-back pain sometimes spreading to the back of his epigastric region was not resolved by NSAID analgesic therapy. Interestingly, his chief complaint is a frequent so-called “atypical” symptom of MI [5]. Coronary angiography revealed 95% critical stenosis in the LCx, 30% stenosis in the mid-LAD, and non-significant stenosis 30% in mid-RCA. He was managed successfully with primary PCI. Studies indicate that symptoms labeled as “atypical” pain concerning the likelihood for MI receive delayed treatment and have a poorer outcome. Atypical pain is frequently defined as epigastric or back pain or pain that is described as burning, stabbing, or characteristic of indigestion [5]. Typical symptoms usually include chest, arm, or jaw pain described as dull, heavy, tight, or crushing. DeVon *et al.* [5] propose no longer using the terms typical and atypical symptom assessment for MI so that proper and rapid diagnostic testing can be undertaken. Mahajan [6] on his paper state that adults in the United States remain unaware of the symptoms of MI. Back pain is frequently associated with neurological disorders. But in neurologic disorders often comes with neurological impairment either paralysis or paresthesias. Not only that, other neurologic special examinations can also be performed to exclude neurologic diagnoses. Patrick *et al.* [7] mentioned that there are so many differential diagnoses for back pain and a good history and physical examination are required to get the right diagnosis.

The ECG of this patient revealed an evolution of ischemic signs. The first ECG showed inferior OMI, complete RBBB, and first-degree atrioventricular block. The symptoms had worsened by day 3 of hospital admission, with inverted T waves at II, III, aVF, and V3–V6. After referral to our facility, we noticed a sign of a new inferior anterolateral ischemia. According to Cooper *et al.* [1], upright T waves at V1 may suggest posterior ischemia. Widimsky *et al.* [8] termed upright T waves at right precordial leads “pseudo-normalization” of T waves, and it is an ominous sign of infarct when inverted T waves dynamically become upright. The upright T waves at V1–V2 in conjunction with the RBBB appear as a “mirror-image” of inverted T waves at posterior V7–V9. It could as represent isolated lateral myocardial infarction (LMI) of the circumflex artery, which is usually the artery responsible for infarction [9]. Life-threatening MI might be missed when RBBB is present, and the presence of RBBB should be able to predict a proximal RCA lesion [1].

The right bundle branch runs in the interventricular septum, and the blood supply is the left coronary circulation [10], mostly provided by the first septal branch separated from the LAD. Therefore, new-onset RBBB is likely caused by proximal occlusion of the LAD [11]. Besides the LAD, the right bundle branch also receives collateral circulation from the right circumflex artery or the LCx [12]. The 12-lead ECG is particularly insensitive for LCx occlusion because of the absence of lateral precordial leads and the late depolarization of the lateral wall [13]. Interestingly, in our case, coronary angiography revealed that the culprit artery was the LCx rather than the LAD.

MI can cause direct cellular damage to the right bundle branch. Increased right intraventricular pressure, chronically as in cor pulmonale, can stretch the right bundle branch causing a bundle branch block. RBBB is generally a slowly progressive degenerative disease of the myocardium [10].

Patients with new-onset RBBB may need more attention. Wang *et al.* [11] have shown RBBB may mask the early diagnosis of ST-elevation myocardial infarction (STEMI). Moreover, new-onset RBBB is occasionally caused by acute myocardial infarction (AMI). Thus, a number of patients with ischemic symptoms and new-onset RBBB may suffer from STEMI. The presence of RBBB is a significant independent predictor of a poor prognosis, including higher rates of acute heart failure, complete heart block, and the need for a permanent pacemaker, as well as higher in-hospital mortality [10, 12]. New RBBB is likely to lead to a higher incidence of cardiogenic shock and increased long-term mortality [11].

Non-STEMI and acute coronary occlusion have a mean delay of > 24 h before PCI [13]. There are different treatments to carefully handle RBBB and the presence of an ischemic sign on ECG (an upright T wave at V1). According to Cooper *et al.* [1], cardiac catheterization of a patient whose ECG revealed RBBB and an upright T wave at V1 (as a “mirror-image” of inverted T waves at posterior V7–V9) revealed 80% stenosis of the distal left main artery, 60% stenosis of the mid-LAD, and 95% stenosis at the ostium of the RCA. One day after catheterization, the patient received coronary artery bypass grafting (CABG). Manzur-Sandoval *et al.* [9] reported a patient with non-reperfused MI with RBBB. The coronary angiography showed TIMI Flow 0 at the proximal segment of the circumflex artery, and the patient received initial management with aspirin 300 mg, clopidogrel 300 mg, unfractionated heparin 4000 IU, and atorvastatin 80 mg. The 2017 European Society of Cardiology guidelines state that for AMI management, it may be difficult to detect transmural ischemia in patients with chest pain and RBBB. MI and RBBB have a poor prognosis. Hence, a primary PCI strategy needs to considered when the patients presents RBBB and persistent ischemia symptoms based on the ECG pattern [14].

## Conclusion

It is a challenge for clinicians to recognize and carefully assess a patient’s complaints even if they are admitted with pain that is atypical of MI, and especially if they have a history of metabolic syndrome. When the ECG findings change, clinicians need to pay attention to—and neither underestimate nor ignore—the presence of RBBB because it may contribute to life-threatening occlusion of the left coronary artery. Therefore, it is important to consider the indication of reperfusion in symptomatic patients with RBBB.

## Data Availability

The data that support the findings of this study are available from the corresponding author, upon reasonable request.
